# “Smells like team spirit” the association between running club membership and performance in the London Marathon: An economic analysis

**DOI:** 10.1371/journal.pone.0306853

**Published:** 2024-07-31

**Authors:** Lee-Ann Burke

**Affiliations:** Department of Economics, University College Cork, Cork, Ireland; The Hong Kong Polytechnic University, HONG KONG

## Abstract

This study examines the association between club membership and marathon performance using a dataset of 206,653 London Marathon runners. Our results show a statistically significant association between club membership and marathon performance for both males and females which sees club membership potentially mitigating pace decline with age and resulting in substantial improvements in finishing times of up to 40 minutes. We implement a production function framework and align with three principles of economic organisation. The findings have relevance for marathon participants, coaches, and athletic associations as well as implications beyond athletics to other sports or cooperative activities.

## Introduction

Since the 1970s, there has been a remarkable surge in the popularity of running as a sport [1] which is accompanied by a corresponding increase into research dedicated to its understanding [2]. Evidence on how to run faster, for longer, and more efficiently is published in academic journals, fills internet sites, and sells books and magazines across the world. This paper seeks to add to the already substantial literature in the running field [3–6] by measuring the association of running club membership on marathon pace.

While existing literature examines training techniques, nutrition strategies and other physiological factors [5,7–9] affecting marathon performance, this study aims to advance the research by exploring the relationship between social factors by focusing on running club membership and its relationship to running performance. Our research addresses this gap in a systematic exploration of the correlation between the two. We treat club membership as a variable encompassing everything a running club offers such as, but not limited to, structured running programmes, advice and training from experienced coaches and seasoned runners, guidance on running form, nutrition, hydration and running gear for example. [10–12]. This study aligns with research that being part of a group can increase productivity [13–17] and we use production function theory to analysis this input output relationship. The results of our analysis can be translated into a specific number of minutes potentially shaved off a per-age category average finishing time, a number easily understood by runners and coaches alike. It adds relevant information previously unavailable and therefore enriches the knowledge base of sports science.

The findings from this study will be of interest to both academic and non-academic stakeholders alike for a number of reasons. First, given the economic framework used in the analysis, this research provides strong empirical evidence on the benefits of economic organisation. It serves to bridge the gap between academic and practical applications and, in addition to economics, also has relevance in the disciplines of sociology and psychology. Second, athletic clubs and their governing organisations are provided with evidence of a positive club association with run-times which can inform their recruitment and marketing strategies across all age cohorts. In addition, the results can contribute to evidence-based policies in the field of marathon running. Evidence of the group association could be applied to other individual sports such as cycling, swimming, and boxing which further increases the reach and relevance of the analysis. Third, by providing runners with information regarding the quantifiable benefits of club membership, they may be encouraged to bring about changes in training practices by joining a club. For ambitious runners, faster marathon finishing times across age categories could result in an individual being eligible to apply for a place in some of the more prestigious marathons of the world such as Boston, New York, or Chicago. For recreational runners, joining a club and availing of the expertise and group mentality and increased productivity therein can keep a runner interested in the sport by setting and meeting personal bests and ultimately have it become a lifelong activity resulting in improved performance and numerous health benefits, both physical, mental and social [18–23]. Fourth, by considering the motivations and characteristics of runners, event organisers, tourism authorities and destination marketeers can tailor participant services, advertising campaigns and marketing strategies to attract and accommodate both national and international participants to their races.

## Relevant theory and literature

From the perspective of production function theory, a marathon runner could be viewed as a technical unit that produces an output using a given combination of inputs. The output produced in this case, is the running pace. We would expect or assume that a marathon runner wants to maximise output given the level of these inputs. This paper treats the independent variables as inputs and investigates the inclusion of club membership as a relevant input in the production function. Appling production function analysis is not unique in the sports literature as it offers a structured approach to evaluate how different resources can contribute to the performance of teams and athletes. Productions functions have been applied in the areas of basketball, cricket, swimming and water-polo [24–28] with outputs relevant to the specific sport such as game attendance, number of wins, win percentage or time to complete a race We investigate the benefits of group cooperation as they relate to productivity increases across a variety of published areas and then apply this idea to marathon running in the empirical analysis.

To further explore the benefits of group membership [13,29,30], and justify their place in a production function, we focus mainly on three of the ten universal principles outlined in Stoelhorst and Richerson’s [31] theory of economic organisation and discuss these principles in a running club setting. Primarily (Principle 1), humans are social animals who instinctively form groups with the aim of increasing cooperative behaviour. A running club can facilitate cooperation among runners by fostering a sense of community, shared norms and mutual support. We also learn from the more successful members of the group (Principle 4) and, on acquiring this tacit knowledge, our deference towards these successful members increases. This has a positive effect on our actions as our behaviour changes to imitate the behaviour of successful member and their actions. Henrich and Gil-White [32] refer to this phenomenon as *prestige hierarchy*. Principle 7 is also applicable as it outlines the dual nature of groups; social and economic. Cooperation between members being sustained relates to the social nature while competing with other organisations (such as other running clubs) is economic in nature as is relates to competition for scarce resources (such as a podium position or a higher ranking in a specific category, for example). These three principles combined can help to further our understanding of how human behaviour and group dynamics can result in an increase in runners’ productivity and can justify its inclusion in an output analysis.

Empirical evidence on how group interaction can increase productivity in sport is evident in the literature [13,15,30,33–35]. On how groups make better self-interested decisions, Charness and Sutter [13] explain that being a member of a group can help individuals overcome issues around procrastination and reduced productivity, both of which can result in sub-optimal results. They present examples from several different areas such as the workplace, the education and exercise arenas and show that individual productivity increases are often seen when individuals are working as part of a group [13]. In a study on gym participation, Condliffe et al. [30] report a sustained increase in gym attendance when members are part of a team and, when members are made aware of the performance of their teammates, within team competition increases resulting in better individual outcomes. A study using data from ELSA (English Longitudinal Study for Aging) [34] reports that sustained physical activity into old age is more likely if the individual is a member of an exercise or sport group. Indeed, respondents not members of a sport or exercise group had 1.27 times the odds of dying across the 10-year follow up period compared to their counterparts with membership. The benefits of teamwork also go beyond the sporting world. Studies also show that being part of a group can affect areas such financial decision making [14], study [15], research through co-authorship, [13], productivity of workers in a supermarket chain with the introduction of higher productivity co-workers [16], weight loss [36], and envelope stuffing [37].

Lev and Zach [38] suggest that running can become integral to a person’s identity, a sentiment also shared by Shipway et al. [29] who emphasize that runners develop a strong emotional connection to their group and the broader running culture through social interactions and the exchange of running techniques within their community. In addition to the tacit knowledge shared in clubs, they also foster a sense of belonging, community, sharing of experiences and social fulfilment. Jane, [27] while not directly discussing swimming club effects, highlights that peer performance in swimming races is an important indicator of the performance of an individual swimmer.

In addition to the productivity benefits reported, there is also evidence that the size of the group can result in more productive outcomes. Franken et al. [3] discuss a Core Sports Network (CSN) which is a network of sporting ties who play an important role in running as an activity. The greater the size of the CSN the more likely the individual at the core is to receive social support. Not only does the size of the CSN affect running frequency but also increases the attendance at running events. Drawing evidence from the non-sporting arena, Gaughan et al. [39] include the size of the network in addition to its composition with respect to outcomes in academia. They note that larger networks can be better resourced, and, in the models incorporated, for each additional member in network size, a two percent increase was seen in scholarly activity. Size was significant across several models run. In a similar sphere, Besancenot et al. [40] investigate the effect of network size and quality on academic productivity as measured by an individual academics citation scores single author publications and find, contrary to their hypothesis, that size of the network is insignificant but quality of the network remains important.

### Additional marathon performance factors

The existing body of literature extensively explores the influence of age and gender on sports performance, including marathon running and other sporting disciplines. It is widely acknowledged and empirically supported that age and gender exert notable effects on athletic outcomes [41–44]. This understanding is reinforced by the widespread practice observed in races globally, where participants are categorised according to age groups during the application process. Upon completion of the race, results are typically disseminated by overall placement, placement by gender, and placement within specific age categories. Certain biological differences such as lower body fat, greater muscle mass, larger hearts, greater haemoglobin concentration for example [44] can result in faster finishing times for males. Factors such as breast size and menstrual cycle can further increase the finishing time differences between males and females [4,45].

In our analysis, while analysing non-GBR participant data, we aim to explore the concept of running tourism and report the potential differences in pace between sports tourists and local runners. The aspect most important to local runners can be the race result achieved whereby running tourists exhibit a different range of motivations such as sensation seeking, event image, destination personality and enriching the running experience [46–49] and much of the research focuses on these motivations. Due to data limitations, exploration of motivation is not feasible in this study however we seek to add to the existing literature on sports tourism by reporting on pace differences between both sets of competitors, information on which is not wholly evident in the literature.

According to Vernon et al. [50], the weather is a contributing factor in marathon performances. Using London Marathon 2018 data, they report that for every five degrees Celsius increase in temperature is associated with a 2.6% decrease in finishing times and outlines the pacing changes required given an increase in temperature and a desire to achieve a particular running time. Knechtle et al. [51], in their study on Boston Marathon races from 1972 to 2018, provide further evidence of temperature increases on the Boston Marathon finishing times. Indeed, other weather occurrences like wind and pressure had a negative effect on performances of athletes including elite athletes. Environmental factors such as pollution are the focus of Guo and Fu [52] with an analysis of 0.3 million marathon runners over a two year period in China. Their paper refers to running as short run productivity and that air pollution has a negative effect (air pollution elasticity on finish time of 0.0408) on runners’ performance. In general, there is a large body of evidence citing the negative effects that weather, such as high temperature, can have on marathon performance [6,53–56].

Given, however, that running is not exclusive to any specific demographic, there is no single prediction of performance equation that can be applied by any one individual, as discussed in Keogh et al. [9] in their systematic review of 144 equations from 36 running-related studies. There are a substantial number of additional inputs, uncontrolled for in this paper, that could determine marathon performance, some of which include individual characteristics, training and recovery techniques, fitness wearables, footwear and nutrition.

We operate under the assumption that marathon runners generally possess a certain level of motivation. This assumption arises from the description of a marathon as being categorised as an endurance sport [8,57–60] and evidence suggesting that a high level of motivation is needed in order to partake in endurance sports [61–63]. The significant presence of the characteristic in this field has warranted the creation of the Motivation of Marathoners Scale (MOMS) [64]. This scale has become a cornerstone in understanding the diverse motivations which drive individuals to participate in this endurance event. Further discussion of the various motivations that are experienced by marathon runners can be found in [48,49,65–70]. Furthermore, we assume that current and potential marathon runners, driven by their motivation, could be receptive to evidence suggesting that running with a club is correlated with enhanced performance, as explored in this study.

In conclusion, given the literature cited, there is evidence, both theoretical and empirical to support the inclusion of club membership; a holistic variable which encompasses a wide variety of components such as collective training, commitment and overall team spirit, into a marathon output production function analysis as its inclusion could enable a runner to maximise their productivity and increase their running output as measured by running pace.

## Data and methodology

The data used in this study is from five London Marathons from 2018 to 2023, results of which have been scraped from the London Marathon Website [71]. From 2021 onwards the London Marathon also has a virtual component; these data are not included. The London Marathon is widely known as one of the worlds six major marathons, along with Chicago, Boston, Berlin, New York City and Tokyo [55]. There are 206,653 observations (runners) in our data after elite runners and non-finishers have been excluded. A place in the London Marathon is highly sought after and over 400,000 runners applied for a place at the 2023 race and 2024 entries surpassed 500,000. The data provides information on gender, age category, country, club name, overall place, overall place in age category, split time, and overall time. From this we calculate a ‘minutes per kilometre’ variable, which will be used as the dependent variable.

### Dependent variable: Minutes per Kilometre, MpKM

**Fig 1:**
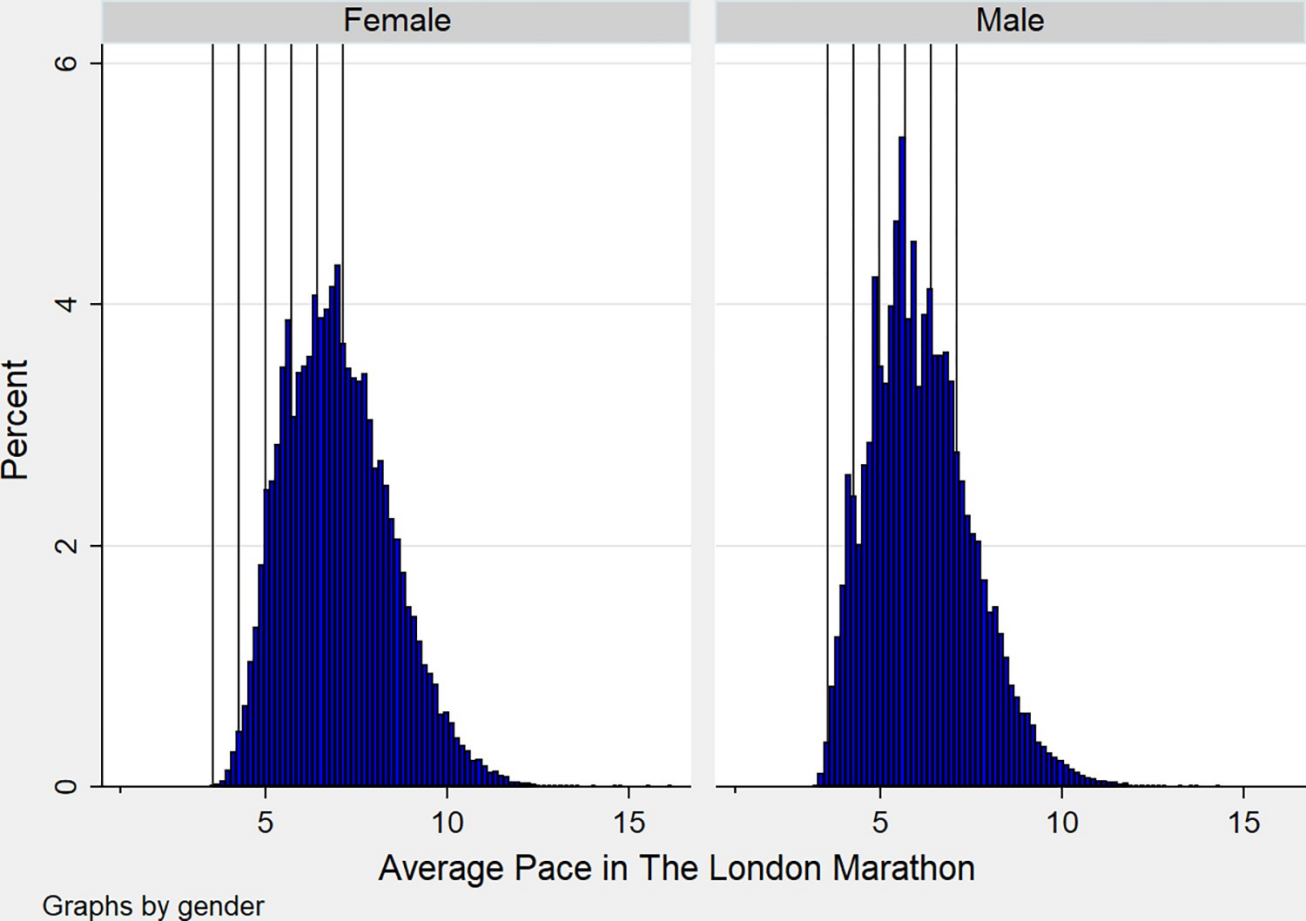
Distribution of minutes per kilometres in London Marathons 2018–2023. Reference lines(L-R): (1) Sub 3.5 hrs, (2) Sub 4 hrs, (3) Sub 4.5 hrs, (4): Sub 5 hrs.

We construct a pace variable as our measure of performance, with lower values measuring higher performance. Total chip time taken to complete the route is divided by the kilometre distance of a marathon, i.e., 42.195km. The data are reasonably normally distributed, (see Fig 1 below). In Fig 1, we have also included finishing times at 30-minute intervals, as indicated by the reference lines, from 3.5 hours to 5 hours, as these are well known target finishing times in marathons. At many of these points, for all runners, we can see an increase, or a spike, referred to as bunching [72], in the proportions of runners completing their marathon and interestingly, that increase is generally higher in male runners.

### Independent variables

The variables included in the analysis are presented in Table 1A and 1B below. Over the course of the five years of data we can see that the average pace, apart from that reported in 2018, has remained relatively consistent. In 2018 the pace reported translates into an average finishing time of 4h 53m whereas average finishing time in 2023 is 4h 27m. The gender breakdown is relatively consistent over the period and comparable to other large marathons. Age is reported in categories and given the smaller numbers in the older age categories; these have been merged into the 65+ age group. Over the course of the five years, there has been a fall of almost six percentage points in the proportion of runners in the 18–39 age category and an increase in runners over 45 years of age indicting a slight shift upwards in the average age of participants.

**Table 1:** a. Variable names, descriptions and means. b. Further Breakdown of Group Size per Year.

Name	Description	2018	2019	2021	2022	2023
MpKM	Average Minutes per KM (Pace)	6.935	6.401	6.387	6.541	6.321
Gender	1 if Male, 0 = Female	0.580	0.582	0.598	0.584	0.590
Club	1 if Club member, 0 otherwise	0.287	0.290	0.092	0.175	0.171
18 to 39	1 if between 18 and 39 years old	0.505	0.492	0.441	0.443	0.462
40 to 44	1 if between 40 and 44 years old	0.172	0.167	0.170	0.163	0.160
45 to 49	1 if between 45 and 49 years old	0.141	0.148	0.152	0.147	0.141
50 to 54	1 if between 50 and 54 years old	0.098	0.097	0.116	0.121	0.120
55 to 59	1 if between 55 and 59 years old	0.047	0.053	0.065	0.070	0.067
60 to 64	1 if between 60 and 64 years old	0.023	0.026	0.033	0.035	0.035
65 & over	1 if 65 years old and over	0.015	0.017	0.022	0.022	0.020
Group-size	Participants per club (1-n)	112	105	69	119	105
non-GBR	1 if non-GBR Registered	0.146	0.196	0.169	0.271	0.268
n		39,095	42,560	35,870	40,619	48,509
**Year**	**Observations**	**Mean**	**Std. Dev.**	**Min**	**Max**	
2018	11,204	13.60	16.61	1	112	
2019	12,344	13.72	18.06	1	105	
2021	3,310	8.20	10.94	1	69	
2022	7,105	13.54	18.24	1	119	
2023	8,301	15.43	19.15	1	105	

A binary club membership variable was created indicating that a runner is part of a club. We can see that in both 2018 and 2019, close to 30 percent of runners had registered using club information. This fell in 2021, as expected, due to the Covid 19 pandemic and the operations of some clubs being on hold. 2022 shows that club members are back up to just over 17 percent. Furthermore, from this data, we have generated a variable called ‘Group Size’ which is a count of the number of runners from each participating club 1,934 recognised clubs participated with Group Size ranging from 1 to 119 While we acknowledge that Group Size may not perfectly correlate with the size of the running club, we assume a high degree of correlation given that larger clubs are more likely to have more runners competing in the event. In any year, on application, a runner selects their club from a drop-down menu. However, the manner in which the club’s name is displayed varies at times across the five races. To address variations in how club names appear in the data across time, the names are standardised by club to ensure an accurate count of runners from each club and ensure data consistency. A binary variable named ‘non-GBR’ was created to indicate if the runner had registered their country as other than GBR. Our objective, in the interpretation of the results, is to view the coefficient as indicative of running tourism. In 2022 we see a more than a ten-percentage point increase in ‘running tourists’ in the race with that proportion continuing in 2023.

A significant proportion of runners registered their running club’s name as ‘Other’. While we do categorise these runners as being a club member, we are unable to include them in the list of club names used to generate the group size variable as we cannot assume interaction between these members. Almost two thirds of those selecting ‘Other’ as their club’s name could be classified as running tourists as they are non-GBR runners. The ‘group size’ variable counts the number of runners representing each of the clubs across each of the five years. Group size reaches a maximum of 119 in 2022 and the average group size ranges from 8.2 in 2021 to 15.43 in 2023.

**Fig 2:**
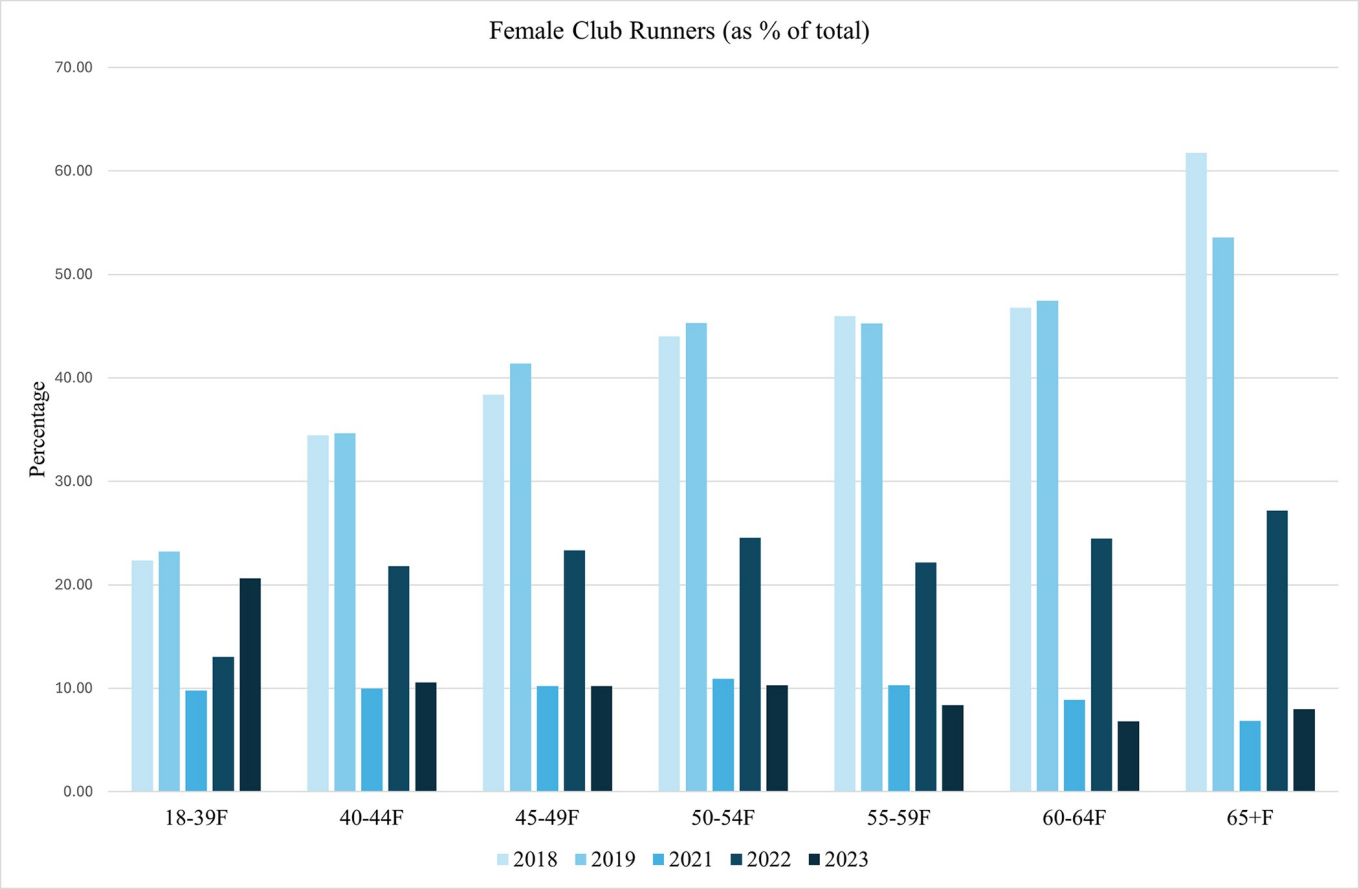
Club members as a proportion of total runners per age category: Females.

**Fig 3:**
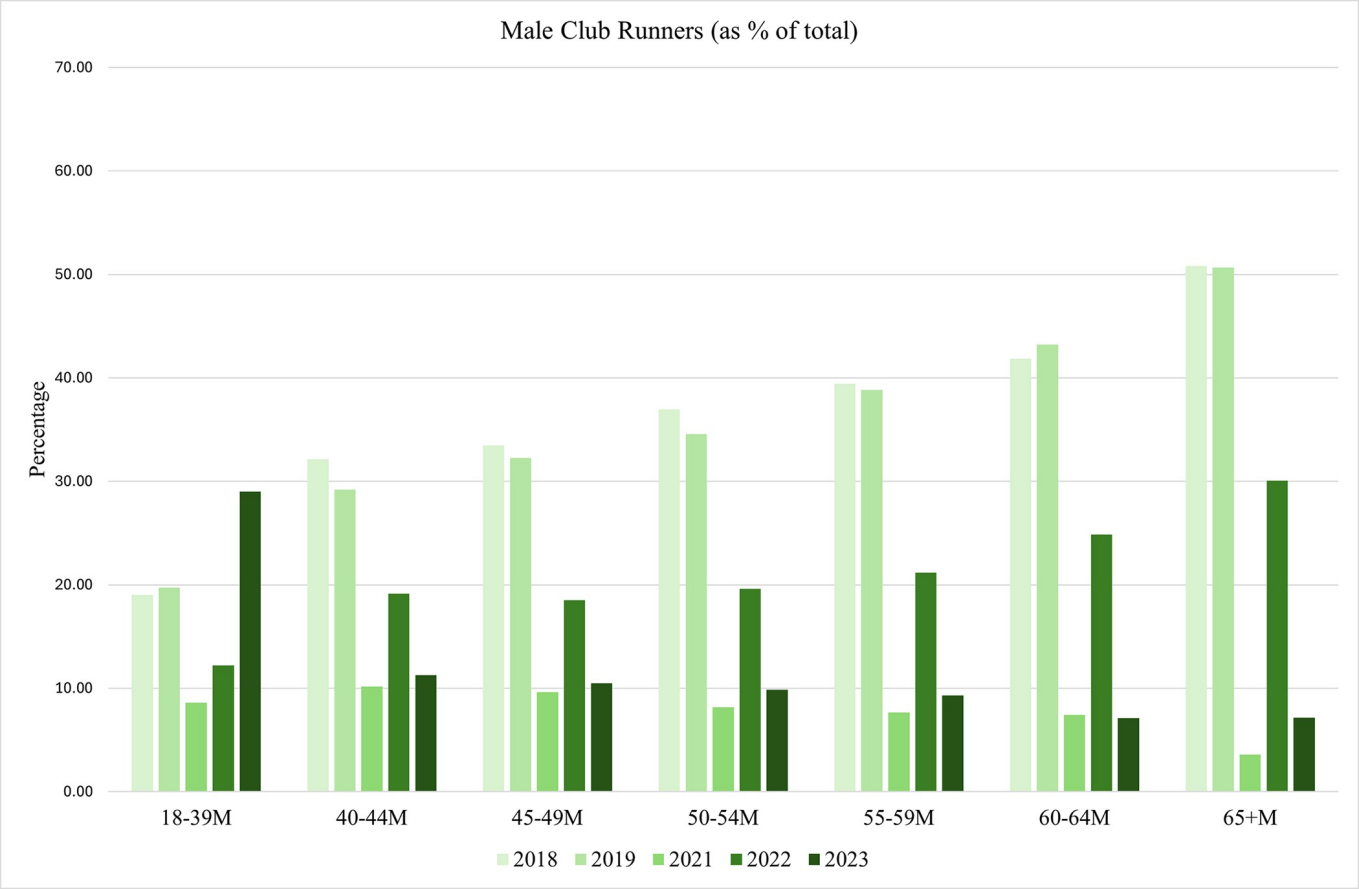
Club members as a proportion of total runners per age category: Males.

The proportion of club members in all age categories are displayed in Figs 2 and 3. We have seen, from Table 1A, that as age increases participation rates in the London Marathon decrease. What we can see in Figs 2 and 3 however, is that prior to 2021, the older the runner, the more likely that he or she is a club member. In 2018 for example, just over 20 percent of females between 18 and 39 are club members but club membership rate at least twice that in females over 50. We also see similar patterns for males although across most of the data we see males less likely to be a club member compared to females. From 2021 onwards, the data is telling a different story with smaller proportions of club members in older age categories. This may, in part, be due to the increased likelihood of Covid19 infection in older cohorts and a reflection of increased risk-aversion as people age.

Table 2 presents a breakdown of pace (minutes per kilometre) across the age categories, gender, and club membership. In addition, we have included the average finishing times per each category also. As expected, we find that, in general, pace slows as age increases and males record a faster pace than females across the same age categories. However, across all age categories and for both males and females, being a part of a running is associated with a quicker pace. For males this club association is strongest for those under 40 and the absolute difference in pace decreases with age. These differences fluctuate slightly for females. It is interesting to note that in the 18–39 age group, female club members record a slightly higher pace, on average, than non-club member males, indicating that club membership for this category could negate the gender effect. This pace difference, when average finishing times are considered, translate to about two minutes faster on average.

**Table 2:** Breakdown of average mins per KM (*finishing times*) by gender, age category and club membership.

Age	Males Non-Club	Males Club	Females Non-Club	Females Club
18 to 39	6.30 (*4hrs26*)	5.15 (*3hrs37*)	7.11 (*5hrs00*)	6.26 (*4hrs24*)
40 to 44	6.15 (*4hrs20*)	5.40 (*3hrs48*)	7.04 (*4hrs57*)	6.53 (*4hrs36*)
45 to 49	6.24 (*4hrs23*)	5.57 (*3hrs55*)	7.05 (*4hrs58*)	6.61 (*4hrs39*)
50 to 54	6.37 (*4hrs29*)	5.81 (*4hrs05*)	7.20 (*5hrs04*)	6.77(*4hrs46*)
55 to 59	6.56 (*4hrs37*)	6.05 (*4hrs15*)	7.30 (*5hrs08*)	7.00 (*4hrs55*)
60 to 64	6.73(*4hrs37*)	6.22 (*4hrs22*)	7.41 (*5hrs13*)	7.09 (*4hrs59*)
65 & over	7.29 (*5hrs08*)	6.90 (*4hrs51*)	7.83 (*5hrs30*)	7.59 (*5hrs20*)

### Econometric model

In our analysis we employ an OLS econometric technique to examine the relationship between club membership and pace, as measured by minutes per kilometre (MPKM), while also considering the impact of age. We recognise that age is associated with a decline in pace, and we hypothesize that the association of club membership may decline across age groups. In order to capture this potential interaction, we include an interaction term between club membership and age group in our model. In addition, we control for club size, running tourism and year. Our OLS robust regression model is shown in Eq 1 and all statistical analysis was carried out using Stata 14.


$$y_{i}=\beta_{0}+\beta_{1}{{clubmember}_{i}*\beta_{2}AgeGroup}_{i}+\beta_{3}{Teamsize}_{i}+\beta_{4}{Teamsize}_{i}^{2}+\beta_{5}{NonGBR}_{i}+\beta_{6}{Year}_{t}+u_{i}$$ Eq 1

Where *y* is MpKM (minutes per kilometre), *i* is individual *i*, *u* is the error term, *β*_0_ is the intercept and *β*_1_ *to* *β*_5_ are slopes of the explanatory variables. Specifically, *β*_1_ * *β*_2_ captures the interaction effect between club membership and age group.

## Empirical results

The results of the robust OLS repeated cross-sectional regression and subsequent marginal analysis are presented in Table 3. Columns (a) report the OLS coefficients where the reference category is runners aged between 18–39 who are not running club members, (b) report the marginal effect of club membership for each age category and (c) convert the marginal effects to improvement in overall marathon finishing times. All results are reported in hundredths of a minute. To provide a more comprehensive analysis, the results are reported by gender.

**Table 3:** OLS regression and marginal analysis results.

	Females			Males		
Variable	(a) OLS Coefficient	(b) Margins: Overall Pace	(c) Improve Time (mins)	(a) OLS Coefficient	(b) Margins: Overall Pace	(c) Improve Time (mins)
Age * Club Member						
*18 to 39 # No Club*	*reference*	7.05		*reference*	6.23	
18 to 39 # Club	-0.723***	6.33***	30.38	-0.969***	5.26***	40.93
40 to 44 # No Club	-0.003	7.05		-0.074***	6.15	
40 to 44 # Club	-0.532***	6.52***	22.36	-0.819***	5.41***	31.22
45 to 49# No Club	0.054***	7.10		0.041***	6.27	
45 to 49# Club	-0.438***	6.61***	20.68	-0.657***	5.57***	29.54
50 to 54 # No Club	0.225***	7.27		0.197***	6.43	
50 to 54 # Club	-0.265***	6.78***	20.68	-0.419***	5.81***	26.16
55 to 59 # No Club	0.377***	7.43		0.415***	6.64	
55 to 59 # Club	-0.035	7.01***	17.72	-0.160***	6.07***	24.05
60 to 64 # No Club	0.507***	7.56		0.612***	6.84	
60 to 64 # Club	0.056	7.10***	19.41	0.013	6.24***	25.32
65 + # No Club	0.917***	7.97		1.163***	7.39	
65 + # Club	0.528***	7.58***	16.46	0.653***	6.88***	21.52
Group size	-0.017***			-0.018***		
Group size^2^	0.0001***			0.0002***		
Non GBR	-0.534***			-0.422***		
2019	-0.500***			-0.528***		
2021	-0.684***			-0.744***		
*2022*	-0.417***			-0.491***		
*2023*	-0.655***			-0.653***		
*Intercept*	7.643			6.846		
n	85,484			120,807		
Adjusted R^2^	0.093			0.136		

*Notes*. All results are reported in 100ths of a minute.

* *p* < 0.1

** *p* < 0.05

*** *p* < 0.001.

The fastest age cohort of runners are those aged between 18 and 39 with running pace decreasing with age thereafter. All non-club runners, apart from those age 40–44, are slower in pace than their younger counterparts with pace slowing progressively with age and in most cases, being slightly more pronounced for males.

Regarding club membership, female club runners aged between 18 and 39 are, ceteris parabis, about 43 seconds faster per kilometre (60*-0.723) than non-club runners in the same age category. For males of the same age, club runners report a 58 second per kilometre improvement in their marathon pace. Of particular interest is that these statistically significant coefficients are seen up to and including the 55–59 age category for men and up until the 50–54 age category for women. The pace for all categories under 60 years of age is quicker when club membership is included. The absolute values on the beta coefficients decrease as we progress through the age categories to the degree of an improvement of about fifteen seconds per kilometre for women in the 50–54 age category and about twenty-five seconds improvement for males when compared to those age 18–39 not in a running club. Indeed, for club member males aged between 55–59 the benefit is approximately 10 seconds improvement per kilometre.

Also, there is no statistically significant difference in running times (just over two seconds; 60*-0.035) between women aged between 18–39 who are not club members and women between 55–59 who are club members, all other things being equal. The association of pace and club membership become progressively less pronounced with age and are larger for males (range for females is -0.723 to 0.528 and for males is -0.969 to 0.653). Essentially, the results show that the well documented age effect seen in running is negated by club membership for a significant period of time and across most age categories. It is only in the over 60 age categories that the coefficients are flipped from negative to positive indicating slower pace.

The results for a club’s group size participating in the London Marathon reveal a statistically significant relationship between group size and performance. Specifically, each additional runner affiliated with a particular club is associated with an improvement in pace of approximately one second, for both male and female runners. However, when introducing a quadratic term for group size (group-size^2^), the impact of group size on performance diminishes as the group size increases.

Each year is included as a dummy variable to control for differences in weather. All years report a negative statistically significant coefficient when compared with 2018 with the pattern between males and females being quite similar. Our results also show that both male and female running tourists run a quicker pace than their local counterparts with the relationship between running tourism and pace being greater for females. The statistically significant coefficients of -0.534 and -0.422 translate to a pace improvement per kilometre between GBR and non GBR runners of 32 seconds and 25 seconds approximately for females and males respectively.

Implementing marginal analysis allows us to express the pace differences within age groups as improvement in finishing times across all the age groups. We can see, (Column b), that the largest pace improvements are seen in the younger age groups with males exhibiting a greater pace improvement than females at 0.97 minutes compared to 0.72 minutes. The pace reduction across the age groups, while not linear, is relatively consistent and we find evidence at the 1% significance level that the predictive margins differ for club and non-club members. When these results are translated into finishing time improvements, (Column c), by multiplying the difference between club and non-club running pace by 42.195, we get a true appreciation of the benefits that are associated with running club membership. Females club members aged between 18–39 could, all else considered equal, cross the finishing line approximately 30 minutes sooner than non-members of the same age. For males, a 41-minute improvement in running time could, on average, be expected. For those over 65 years of age, an improvement of 16 and 22 minutes for females and males respectively is possible. While there is still a significant association between club membership and finishing times for older runners, the degree of association is not as pronounced.

## Discussion

The findings of this study provide evidence to support our hypothesis that running club membership is associated with an increase in marathon pace productivity. In addition, our analysis reveals a noteworthy interaction between age, indicating that the benefits of club membership may exist beyond pace enhancement. Club membership may not only negate the adverse effect of age on marathon pace but could help to surpass the performance of younger non club runners. This may mean an increase in competitive years for runners.

It is acknowledged that tangible and measurable marathon performance factors can occur in running clubs, such as specific training schedules, running advice, scheduled runs, and education around nutrition for example [10–12] and we expect that these inputs may have driven the significance of the results. Captured, however, are intangible benefits and social factors that can exist in a group setting, such as the individual motivation and increased productivity [13] when training with a cohort of like-minded and emulated individuals [32], the accountability felt when training for a long race and the kudos received when achieving and sharing personal bests [73]. The club membership association uncovered in this paper, as seen in a range of statistically significant coefficients up about the age of about 60 years old, therefore captures all of these tangible and intangible benefits in a holistic approach. The results therefore provide an argument in support of joining a club to increase marathon performance.

By viewing marathon running as a production process, we can incorporate the concept of the production function in this paper. Using this approach allows us to model and analyse how a range of inputs have interacted and are associated with the increased output of faster marathon pace. Furthermore, the results are supported by strong theoretical underpinnings arising from three principles of economic organisation set out in Stoelhorst and Richerson, [31]. The application of economic theory to a sport related empirical setting further allows researchers to analyse sports phenomena through the lens of economic principles and concepts which support a systematic and analytical approach to the study of sports. In addition, applying the production function in sports related research quantifies the relationship between inputs and outputs which can help to facilitate data-driven decision making both from the perspective of a running club hoping to attract new members and for individual runners hoping to achieve a specific outcome in their running endeavours.

By incorporating marginal analysis into the results, we can interpret our findings in language that may resonate more strongly among the running community. Runners do not typically refer to their efforts and achievements with respect to marathon completion in terms of pace but rather in terms of finishing times. Phrases such as “sub-three” and “sub-four” are commonly used among runners and signify a particular aim when training for and running a marathon. Moreso, these are motivational markers and help runners set specific racing strategies. Indeed, visual analysis of the data (Fig 1), would strongly indicate that this is the case. Knowledge that there are significant gains to be made by joining a running club, in the form of up to 40 minutes for younger men for example, mean that these running times could be achievable and are within reach, especially with the support and resources provided by running clubs. Considering the results in this format increases the accessibility of the findings and can be used by athletics clubs and their organising bodies to recruit new members across all age groups.

The practical implications of these results could include increased motivation for joining running clubs, improved training strategies, the harnessing of community support and, ultimately, race performance improved. The results could be of relevance to those runners who are considering an application to a marathon based on qualifying time such as Boston or New York as a reduction of over 30 minutes for females aged between 18 and 39 for example, is significant and chances of success could be greatly increased when specific time-based goals are achieved. Running clubs, in general, have websites and/or a social media presence. The findings of this study could be advertised via these sources in order to attract new runners and inform existing members of the quantifiable benefits of becoming and remaining a member.

Overall, our results very closely align to previous research on outcome determinants such as age, gender and group size. For example, findings that females aged 60–64 who are not club members are about a half a minute slower per kilometre than those non club members aged 18–39 whereas males of the same age run about 36 seconds slower per kilometre than the youngest group of non-club member males are not unexpected [41–43,74]. The pace of runners is positively associated with the number of participants from a particular club. These findings are consistent with prior research establishing a positive influence of increased cohort size on productivity [3,39].

The inclusion of the year variable, enabling a discussion on the weather during the London Marathon, also aligns with previous research analysing the effect that can the weather can have on marathon performance [6,50,51,55]. The 2018 London Marathon was reported as being the hottest London Marathon on record [50], coming only two years after the coldest (10.2 degrees Celsius) on record. According to the Met office UK, temperatures soared to 24.1 degrees Celsius. 4.5 Litres of water were distributed per person as well as the provision of ice and shower stations along the route and runners were advised against fancy-dress costumes. In subsequent years however, the weather was much milder and more characteristic of that time of the year, with temperatures between approximately 14 and 17 degrees Celsius. The positive jump in pace in 2021 may, in part, be due to the first London Marathon in 2.5 years (889 days) and the resulting anticipation and atmosphere which could have led to a quicker pace overall. There has been no significant change in the route of the marathon over the period.

According to Aicher et al. [75] running tourists might perceive an event differently or have different motivations for applying. These results might suggest that the runner characteristics may ultimately feed into successful running outcomes such as we see reported here. We noted earlier that we interpret the non-GBR coefficient using a running tourist narrative. While not without its limitations, our results show that both male and female running tourists run a quicker pace than their local counterparts. Unlike the other results presented here in the paper, the correlation with pace is greater for females compared to males with female running tourists improving their pace by 37 seconds per minute compared to the 32 second improved in the pace of male running tourists. The results may feed into advertising campaigns by event organisers and tourism bodies to encourage increased participation by running tourists in future events. Increased tourist attendance should also feed into the local economy.

While our study identifies a correlation between club membership and performance output it is important to acknowledge the limitations in inferring causality solely from this association. With the available data, we have controlled for age and gender, but we recognise that other unmeasured variables may influence the relationship. We also acknowledge that bias may exist as club runners could be intrinsically different to non-club runners. We recognise the possibility that clubs may attract more motivated runners. However, it is likely that this bias is mitigated somewhat as previous research shows that those who train for, enter, and complete marathons are, we assume, all motivated and are likely to take any opportunity afforded to them to improve performance. We also understand that reverse causality could be an issue which could potentially influence the observed association of club membership. We are unable to measure the training differences across running clubs however, we assume that a goal of running clubs is to improve runners’ performance. We have acknowledged that there are a wide range of marathon performance indicators present in the literature such as training and recovery techniques and nutrition and hydration strategies amongst others. These are not accounted for in this paper given the data availability from London Marathon. However, we assume that differences in running performance factors are randomly distributed. Given the large sample size, we believe this is a reasonable assumption. In addition, we recognise that we are highlighting an associative and not causal relationship between club membership and marathon performance. We acknowledge that being a member of a club may not directly result in a runner’s increased speed. Further work in this area could include longitudinal analysis of runners which tracks their transition to running club membership as well as employing techniques to suitable data such as propensity score matching. Both techniques could uncover a causal relationship. Lastly, we assume that runners do select their clubs when registering for a marathon, potentially to avail of club guaranteed entry, which, in 2023, were undersubscribed [76]. The decision-making process behind club selection remains unexplored.

While these limitations underscore the need for caution in generalising the findings, they also present opportunities for further research in this area. Despite these constraints, we have made significant findings in relation to club membership, additional evidence on age effects and some interesting findings on club size and sport tourism which we believe add to the ever-growing body of literature and evidence on marathon performance factors and act as a starting point to future research potentially investigating a causal relationship.

## Conclusion

In this empirical analysis, we have employed production function theory to support the inclusion of club membership in an analysis on marathon performance. Our study examines a substantial sample of over 206,000 marathon runners who participated in the London Marathon from 2018 to 2023. In addition, by considering the principles outlined in Stoelhorst and Richerson [31] we acknowledge that individuals possess an inclination to form social connections and engage in collaborative endeavours to achieve common goals and that by doing so increases their productivity and thereby a quicker pace and resulting finishing time.

Through the application of econometric analysis techniques, we have estimated a relationship between club membership and marathon performance for both male and female runners. This association remains statistically significant until almost the age of 60 for males and 55 for females counteracting the typical decline in pace associated with aging. Notably, the club membership association is slightly more pronounced among males. Our findings contribute to the extensive body of research examining the measurable influences on marathon performance, providing additional insights into this area of study.

The knowledge of the strong club membership association holds valuable implications, particularly when considering applications for large marathons worldwide that impose age and gender-specific qualifying times. Our findings offer performance insights for both current and future marathon participants, as well as their running club coaches and athletic associations. This understanding can inform individual strategic decisions related to club membership, not only in running clubs but any sporting group or club, with improvements envisaged in performance levels.

We have shed light on the inherent social tendencies of individuals and their impact on marathon performance. Our study contributes to the existing body of research in this field, presenting practical implications for marathon participants and the wider running and sporting community. By leveraging these insights with causal analysis in further work, we can strengthen our understanding of the mechanisms at play between club membership and marathon outcomes.
